# Comparison of Nephrolithiasis Prevalence in Two Bottlenose Dolphin (*Tursiops truncatus*) Populations

**DOI:** 10.3389/fendo.2013.00145

**Published:** 2013-10-16

**Authors:** Cynthia R. Smith, Stephanie Venn-Watson, Randall S. Wells, Shawn P. Johnson, Natalie Maffeo, Brian C. Balmer, Eric D. Jensen, Forrest I. Townsend, Khashayar Sakhaee

**Affiliations:** ^1^National Marine Mammal Foundation, San Diego, CA, USA; ^2^Sarasota Dolphin Research Program, Chicago Zoological Society, Sarasota, FL, USA; ^3^College of Veterinary Medicine and Biomedical Sciences, Colorado State University, Fort Collins, CO, USA; ^4^US Navy Marine Mammal Program, Space and Naval Warfare Systems Center Pacific, San Diego, CA, USA; ^5^Bayside Hospital for Animals, Fort Walton Beach, FL, USA; ^6^Department of Internal Medicine and Charles, Jane Pak Center for Mineral Metabolism and Clinical Research, University of Texas Southwestern Medical Center, Dallas, TX, USA

**Keywords:** urate nephrolithiasis, dolphins, *Tursiops truncatus*, ultrasound, computed tomography, age

## Abstract

In humans, ammonium urate (AU) nephrolithiasis is rare in the Western hemisphere and more common in Japan and developing countries. Among a variety of risk factors, insulin resistance has been associated with urate nephrolithiasis in people. Bottlenose dolphins (*Tursiops truncatus*) are susceptible to AU nephrolithiasis, and it is believed that some populations are more likely to develop nephrolithiasis compared to others. In an effort to better understand population-based risk factors for AU nephrolithiasis in dolphins and their comparative value to humans, sonographic evaluation was performed on dolphins from a managed collection in San Diego Bay, CA (*n* = 40) and dolphins from a free-ranging, nearshore population in Sarasota Bay, FL (*n* = 39) to look for evidence of nephrolithiasis. While 14 (35%) of San Diego Bay dolphins evaluated for the study had sonographic evidence of nephrolithiasis, none of the Sarasota Bay dolphins had evidence of disease. Presence or absence of stones was confirmed by computed tomography in a subset of the San Diego collection (*n* = 10; four dolphins with stones, six without stones). Age was identified as a risk factor, as dolphins with stones in the San Diego collection were significantly older than dolphins without stones (25.4 vs. 19.1 years, respectively; *P* = 0.04). Additionally, San Diego dolphins included in the study were significantly older than Sarasota Bay dolphins (21.3 vs. 13.8 years, respectively; *P* = 0.008). In addition to the previously reported risk factors of hypocitraturia and hyperinsulinemia in bottlenose dolphins, other potential factors include geographic location, managed vs. free-ranging status, prey species, and feeding schedules.

## Introduction

Ammonium urate (AU) nephrolithiasis is known to affect bottlenose dolphins (*Tursiops truncatus*) and can result in azotemia, anemia, hematuria, reduced renal function, and renal loss (1, 2). Kidney stones reported in dolphins have been exclusively composed of AU (3), which is a stone type rarely found in humans in developed countries (4). While it has been hypothesized that some dolphin populations are at higher risk of nephrolithiasis than others, including managed collection vs. free-ranging dolphins, there have been no formal comparisons of stone prevalence between populations (1).

The health of two dolphin groups has been well studied for over four decades (5–8). In Sarasota Bay, FL, health assessments have been performed on wild, free-ranging bottlenose dolphins through a capture-release technique that allows for the safe handling and rapid assessment of individual animals in the population (9). In San Diego Bay, CA, physical exams are routinely performed on individuals in a managed collection of bottlenose dolphins cared for by the US Navy Marine Mammal Program (MMP). Comparisons between these two groups have shown that the studies’ managed dolphins are older; more susceptible to insulin resistance and metabolic syndrome; and have hypocitraturia when compared to free-ranging dolphins (2, 10–12).

Metabolic syndrome and type 2 diabetes have been associated with hypocitraturia, uric acid, and uric acid stone formation in humans (13–18). Due to insulin resistance-associated conditions in dolphins, including fatty liver disease, dyslipidemia, and the higher insulin found in the managed group vs. free-ranging dolphin group, there was an interest in comparing the prevalence of AU nephrolithiasis in the free-ranging Sarasota Bay and managed San Diego Bay dolphins (1, 11, 19).

Ultrasound (US) provides a rapid assessment of organ health in humans and animals, including bottlenose dolphins (20). Studies into pulmonary disease and reproductive status have demonstrated the usefulness of US when evaluating dolphin health (21–24). US has also been used to identify nephrolithiasis in dolphins as well as complications secondary to stones, such as hydronephrosis and urinary obstruction (1). This is not surprising, as kidney stones and urinary obstructions are routinely detected sonographically in humans and companion animals (25, 26).

Computed tomography (CT) has become the first-line imaging modality utilized for the diagnosis of nephrolithiasis in humans (27–29). Unfortunately, CT evaluation on live, free-ranging dolphins is not logistically feasible, as animals would have to be temporarily taken from the wild and transported to a CT scan facility with a wide-bore gantry to allow for the size of an adult bottlenose dolphin. CT has been utilized for the evaluation of health and physiology in live, managed dolphins cared for by the MMP in San Diego, where animals are accustomed to human handling and transport (30–32). Therefore, CT is a feasible option for validation of the US detection of stones in live, managed dolphins.

The objective of the present study was to compare the prevalence of AU stone formation in dolphins with different feeding patterns and prey types. More specifically, we aimed to compare the prevalence of kidney stones between dolphins in a managed collection in San Diego Bay, and a free-ranging, nearshore population of dolphins in Sarasota Bay. US was employed for the detection of kidney stones in both study populations, and animals were characterized as those with stones and without stones. When possible, CT studies were used to validate US results in a subset of the San Diego managed collection.

## Materials and Methods

### Animal care and use

Managed dolphins included in the study are owned and cared for by the MMP in San Diego, CA. The Secretary of Navy Instruction 3900.41G directs that Navy marine mammals be provided the highest quality care. The MMP is accredited by the Association for Assessment and Accreditation of Laboratory Animal Care International and adheres to the national standards of the United States Public Health Service Policy on the Humane Care and Use of Laboratory Animals and the Animal Welfare Act. US and CT exams were performed under one or more of the following conditions: (1) opportunistically during a routine physical exam, (2) opportunistically during a veterinary directed non-routine exam, or (3) predetermined as specified under Animal Care and Use Protocol No. 96-2011, which was approved by a Navy Institutional Animal Care and Use Committee and the Navy Bureau of Medicine.

Free-ranging dolphins were examined under National Marine Fisheries Service Scientific Research Permit No. 15543 (issued to RSW), and IACUC approvals from Mote Marine Laboratory.

### Study populations

A total of 79 bottlenose dolphins were included in this study. The MMP managed collection in San Diego, CA (*n* = 40; ♂ = 24, ♀ = 16) and the free-ranging population in Sarasota Bay, FL (*n* = 39; ♂ = 24, ♀ = 15) had average ages of 21.3 ± 13.1 years (range 3–45 years) and 13.8 ± 9.9 years (range 2–43 years), respectively. All managed dolphins were housed in ocean enclosures in San Diego Bay, CA, and fed fish within the families Clupeidae, Osmeridae, and Scombridae. Fish were inspected and considered high quality, according to hazard analysis and critical control points guidelines for food inspection. The diets of free-ranging dolphins in Sarasota Bay primarily consist of soniferous fish within the families Batrachoididae, Sciaenidae, and Sparidae (33).

### Ultrasound

Renal US examinations were performed in real-time, B-mode either in water or on land. Portable US units (Voluson i, GE Healthcare, Waukesha, WI, USA; M-Turbo or Edge, Sonosite, Bothell, WA, USA) with 2–5 MHz variable frequency and curvilinear transducers were utilized. The GE US unit was equipped with Z800 video glasses (eMagin, Bellevue, WA, USA) and the Sonosite units with Cinemizer video glasses (Carl Zeiss, Oberkochen, Germany) to allow visualization of the US image in sunlight. The sonographer began with the transducer in a dorsal plane and parallel to the spine on the lateral body wall, just caudal to the midpoint of the dorsal fin. A cine loop was acquired as the sonographer fanned through the entire kidney in the dorsal plane, starting dorsally and fanning ventrally. The transducer was then moved into a transverse plane, perpendicular to the long axis of the body, and a second cine loop was acquired while sliding from the caudal to the cranial pole. Additional still images of the kidney were acquired for further documentation when desired. In managed dolphins, both left and right kidneys were examined. Due to time constraints when handling free-ranging dolphins, only the left kidney was evaluated. Following data acquisition, images were reviewed in a radiology viewing room using open-source imaging software (OsiriX, Geneva, Switzerland). Nephroliths were defined as hyperechoic foci with distinct acoustic shadows. Twinkling artifact was not utilized in this study to identify stones.

### Computed tomography

Ten managed collection dolphins were transported to the Naval Medical Center San Diego’s CT facility for examination. Stone burdens were verified either opportunistically if animals were being examined for other health reasons, or purposefully as part of the research study. To ensure adequate hydration status prior to the exam, animals were voluntarily orally hydrated with 1–2 l of fresh water and fed 2.3 kg of fish. Animals either voluntarily beached onto foam pads or swam into stretchers for transport in covered trucks to the CT facility, approximately 20 min away. Upon arrival, sedation was induced with an intramuscular dose of midazolam (range of 0.04–0.08 mg/kg). Animals were placed on the gantry table on a human spine board and gently secured in ventral recumbency with padded straps. Subjects were continuously monitored by veterinary personnel during the exam. Studies were performed using a GE Lightspeed RT 16 helical CT scanner (GE Healthcare, Waukesha, WI, USA) with an 80 cm gantry aperture and 227 kg weight limit for the patient table. Non-contrast helical images were obtained during a normal prolonged end-inspiratory breath hold using contiguous 1.25 mm slices. Following the CT, the animals were transported back to their ocean enclosures and, if needed, were administered a reversal agent at a dose of approximately 0.005 mg/kg flumazenil in the periarterial venous rete (PAVR) of a fluke blade. Following acquisition, images were viewed using open-source imaging software (OsiriX, Geneva, Switzerland).

### Statistical analysis

SAS version 9.2 (SAS Incorporated, Cary, NC, USA) was used to conduct Wilcoxon rank-sum tests to compare ages between the managed and free-ranging dolphins; and, within the managed dolphin group, those with and without detectable nephroliths. Significance was defined as a *P* value less than or equal to 0.05.

## Results

Sonographic evidence of nephrolithiasis was not found in any free-ranging, Sarasota Bay dolphins (*n* = 39; left kidneys only). Nephroliths were detected with US in 35% (*n* = 14) of the managed, San Diego Bay dolphins (*n* = 40; both kidneys examined). Approximately 93% of the stone-forming dolphins had bilateral disease (*n* = 13). Dolphins with stones were characterized as either mild (<5 nephroliths detected) or advanced (≥5 nephroliths detected). Of the dolphins with stones, 29% were mild (*n* = 4) and 71% were advanced (*n* = 10). Stone counts in advanced cases ranged from 7 to 64 total stones per animal, with 47% of stones detected in the left kidney and 53% in the right kidney. Female distribution in the managed collection of animals with stones was 29% (*n* = 4) and in animals without stones was 46% (*n* = 12).

When evaluating left kidneys only of managed collection dolphins, 33% (*n* = 13) of the left kidneys in the managed collection of dolphins had detectable stones. Of these left kidneys, 38% (*n* = 5) had mild stone counts of <5 (ranging from 1 to 4 stones), and 62% (*n* = 8) had advanced stone counts of ≥5 (ranging from 6 to 21 stones). As stated, none of the free-ranging dolphins had detectable stones in their left kidneys.

Computed tomography confirmed the US characterization of all 10 managed collection dolphins examined. Of the 10 dolphins, 4 dolphins were characterized as having stones and 6 as having no stones, using both imaging modalities. For CT evaluation, dolphins with stones were characterized in the same manner as with US (<5 stones, mild; ≥5 stones, advanced). Although CT did confirm the presence or absence of stones as determined with US, there was variability in the classification of mild vs. advanced disease between the two imaging modalities. Of the six dolphins with stones that were evaluated with CT, two changed categorization (one mild to advanced, one advanced to mild) and four remained the same (one mild, three advanced).

Dolphins with stones in the San Diego collection were significantly older than those without stones (25.4 vs. 19.1 years, respectively; *P* = 0.04). Additionally, San Diego dolphins included in the study were significantly older than Sarasota Bay study dolphins (21.3 vs. 13.8 years, respectively; *P* = 0.008), although the overall age ranges of the two sampled populations were nearly identical (3–45 years, San Diego Bay; 2–43 years, Sarasota Bay).

## Discussion

In the current study, dolphins from the managed collection were more susceptible to nephrolithiasis compared to the free-ranging group (35 vs. 0% prevalence, respectively). All kidney stones previously reported from this managed collection have been pure AU (3). Previous studies in managed collections have shown that urinary citrate was significantly lower than free-ranging dolphins (1–3). Here we considered whether age, feeding, and dietary differences between these two populations may lead to lower risk of AU stone formation.

Ultrasound has been previously identified as a valuable diagnostic tool for the diagnosis of kidney stones in dolphins (1). However, CT has rapidly become the first-line imaging modality for the diagnosis of suspected urolithiasis in humans (28). The present study demonstrated that US can accurately characterize stone-formers vs. non-stone-formers, but is limited in its ability to provide accurate stone counts and characterize severity of disease in dolphins, for which CT is superior. However, additional studies with animals examined on land are needed to determine if the US technique could be improved to better characterize severity of stone disease.

Within the managed collection of dolphins, older age was associated with stone formation (25.4 vs. 19.1 years, when comparing dolphins with stones vs. dolphins without stones; *P* = 0.04). In humans, increasing age has not been correlated with AU stone formation. To the contrary, AU urinary stones have been reported more commonly in children 0–9 years than in other age classes (34), and may be attributed to dietary factors and fluid intake (35, 36). Therefore, the correlation of stone formation with increasing age found in the present study is considered unique to dolphins.

Dolphins from this study’s managed collection are at higher risk of insulin resistance and metabolic syndrome, including higher postprandial insulin, glucose, cholesterol, and triglycerides compared to the free-ranging group in this study (11). In addition, this managed collection, albeit with low mortality and high survival rates, is susceptible to metabolic diseases associated with insulin resistance in humans, including fatty liver disease and iron overload (6, 19). In humans, metabolic syndrome and insulin resistance have been associated with uric acid nephrolithiasis (13–18). However, it has not yet been established whether metabolic syndrome and insulin resistance play any pathophysiologic or physicochemical role in the formation of AU stones. In humans, type 2 diabetics share the same phenotypic characteristics including unduly acidic urine (pH ≤ 5.5) which predisposes them to uric acid nephrolithiasis (14, 16–18, 37). An abnormally acidic urine has been associated with defective urinary ammonium excretion and excessive acid production (18, 38).

Seafood diets are typically high in purines, which are metabolized into uric acid, and hyperuricemia has been correlated with seafood and animal protein consumption in humans (39). High purine diets are risk factors for uric acid and AU stone formation, but little research has focused on diets that may specifically increase the risk of AU stone formation. Ketogenic and high protein/low carbohydrate diets have been associated with the increased risk of calcium stones, uric acid, and specifically AU stones in children. The underlying pathophysiologic mechanisms may involve increased ammonium excretion, hypocitraturia, and moderate hypercalciuria due to the provision of acid and purine loads from the diet (40–43).

Another potential mechanism related to the increasing risk of AU stone formation may be related to differences in fish consumed by managed and free-ranging dolphins. Consumption of frozen-thawed fish, which likely has lower water content than the fresh fish ingested by free-ranging dolphins, may add a potential risk factor of decreased water intake. Given the current study’s findings and the potential risks of AU nephrolithiasis from eating high fat, moderate protein, and low carbohydrate diets, there is a need to assess potential differences in the macronutrient and moisture content of diets fed to managed collection vs. that eaten by the free-ranging dolphins.

Low pH is a principal risk factor for uric acid stone formation (18). Physicochemical studies have shown that AU precipitate is the most insoluble urate salt in alkaline environments (44, 45). Previous comparisons between this study’s managed and free-ranging dolphin groups, however, demonstrated no significant difference in urinary pH (6.3 ± 0.08 and 6.1 ± 0.06, respectively) (2). In a single pediatric case study with type 2 diabetes and recurrent nephrolithiasis, consisting of 40% ammonium acid urate and 60% uric acid, nocturnal control of urinary acidity with potassium citrate ameliorated the low urinary pH following administration of alkali during the day (46). Such a diurnal switch in acid-base balance was also previously reported in dolphins (47, 48). Free-ranging dolphins appear to feed frequently throughout the night, and this may help maintain a less acidic state (12). Future studies are needed to explore the diurnal variation in urinary pH, kidney stone risk profiles, and physicochemical parameters in managed dolphins to target an optimal treatment with alkali to reverse these abnormalities during the night time.

The results of this study confirmed that some populations of dolphins are at higher risk than others for AU kidney stone formation, and increasing age is a significant risk factor. US was a useful tool for the rapid diagnosis of nephrolithiasis in dolphins, particularly when CT was not a feasible option in the field for examination of free-ranging dolphins. However US was not as valuable as CT for determining severity of disease or stone burden. Further studies are needed to better understand the impact of diet composition, feeding schedule, and insulin resistance on AU nephrolithiasis in dolphins, all of which are considered potential risk factors for stone formation in dolphins.

## Author Contributions

Cynthia R. Smith conducted and interpreted all US exams and most CT exams, performed data analysis, and composed the manuscript. Stephanie Venn-Watson provided intellectual contributions to the study design and manuscript composition, as well as performed statistical analysis. Randall S. Wells and Brian C. Balmer provided samples from, and facilitated examination of, free-ranging dolphins. Shawn P. Johnson assisted with imaging exams (US and CT) and technique development for managed dolphins. Natalie Maffeo performed the literature review and assisted with manuscript composition. Eric D. Jensen assisted with US and CT examination of managed dolphins. Forrest I. Townsend and Khashayar Sakhaee provided intellectual contributions for the project and resulting manuscript.

## Conflict of Interest Statement

The authors declare that the research was conducted in the absence of any commercial or financial relationships that could be construed as a potential conflict of interest.
